# From across the Seas

**Published:** 1917-02-17

**Authors:** 


### FROM ACROSS THE SEAS.

### New Zealand Doctors Object to Wealthy Hospital Patient.

The New Zealand press, towards the latter part of the past year, gave considerable publicity to the subject of a deputation representing the Tasmanian branch of the British Medical Association which waited on the Premier (Hon. W. H. Lee) and called his attention to the treatment of well-to-do patients in the general hospitals subsidised by the State. Such treatment, the deputation considered, must be regarded as hospital abuse and set up unfair competition with institutions which received no State subsidy. It was maintained that the hospitals in question were intended for people of small means, who were not able to pay a doctor or nursing institution for ordinary treatment, or who required treatment at a reduced rate. It appears that the Launceston Hospital Board, which is chiefly concerned in the dispute, considers that the financial position of a patient should not be the standard by which his admission or rejection is decided; but the medical profession of the Dominion are evidently largely united in upholding the older doctrines of voluntary hospital practice, which are opposed to the views expressed by the Hospital Board. The Premier listened courteously to all the deputation had to say, but adopted a non-committal attitude, and promised that the whole position as regards State-subsidised hospitals would as early as possible be considered by the Government.

### Baby Bonuses in Australia.

From the *Medical Journal of Australia*, Sydney, we learn that the Commissioner of Maternity Allowances has issued returns of claims on the £5 bonus granted and rejected up to the end of June 1915. There were 139,495 claims made in the Commonwealth during the year, and of these 640 only were rejected for non-compliance with the conditions of benefit. The *Medical Journal* evidently does not look upon this method of combating race suicide with altogether approving eyes, and remarks that either the number of fraudulent persons is small in proportion to the population, or the Federal authority is easily gulled. Since the inception of the bonus system in October 1913, £1,781,640 has been paid in claims and £29,728 in administration. A sum of £50 has been paid as rewards for securing convictions in connection with maternity allowance frauds. "Perhaps," says our contemporary, "the reduction of salaries and an increase in the rate for rewards might result in a general saving of public money."

